# Nutritional Strategies for the Individualized Treatment of Non-Alcoholic Fatty Liver Disease (NAFLD) Based on the Nutrient-Induced Insulin Output Ratio (NIOR)

**DOI:** 10.3390/ijms17071192

**Published:** 2016-07-22

**Authors:** Ewa Stachowska, Karina Ryterska, Dominika Maciejewska, Marcin Banaszczak, Piotr Milkiewicz, Małgorzata Milkiewicz, Izabela Gutowska, Piotr Ossowski, Małgorzata Kaczorowska, Dominika Jamioł-Milc, Anna Sabinicz, Małgorzata Napierała, Lidia Wądołowska, Joanna Raszeja-Wyszomirska

**Affiliations:** 1 Department of Biochemistry and Human Nutrition, Pomeranian Medical University, Szczecin 71-460, Poland; ryterska.karina@gmail.com (K.R.); domi.maciejka@wp.pl (D.M.); banaszczak.marcin@gmail.com (M.B.); izagut@poczta.onet.pl (I.G.); zbizcz@pum.edu.pl (P.O.); szpital@szpital-zdroje.szczecin.pl (M.K.); dominikajamiol@interia.pl (D.J.-M.); kldiab@pum.edu.pl (A.S.); 2 Department of Clinical and Molecular Biochemistry, Pomeranian Medical University, Szczecin 70-111, Poland; p.milkiewicz@wp.pl; 3 Liver and Internal Medicine Unit, Department of General, Transplant and Liver Surgery of the Medical University of Warsaw, Warsaw 02-097, Poland; jorasz@gmail.com; 4 Department of Medical Biology, Pomeranian Medical University, Szczecin 70-111, Poland; milkiewm@pum.edu.pl; 5 Department of Diabetology and Internal Diseases Pomeranian Medical University, Szczecin 72-010, Poland; malnap@sci.pum.edu.pl; 6 Department of Human Nutrition, University of Warmia and Mazury, Olsztyn 10-718, Poland; lidia.wadolowska@wum.edu.pl

**Keywords:** NAFLD, NAFLD diet, insulin sensitivity, NIOR, reduction of body mass, fat reduction, liver fat

## Abstract

Nutrients play a fundamental role as regulators of the activity of enzymes involved in liver metabolism. In the general population, the action of nutrients may be affected by gene polymorphisms. Therefore, individualization of a diet for individuals with fatty liver seems to be a fundamental step in nutritional strategies. In this study, we tested the nutrient-induced insulin output ratio (NIOR), which is used to identify the correlation between the variants of genes and insulin resistance. We enrolled 171 patients, Caucasian men (*n* = 104) and women (*n* = 67), diagnosed with non-alcoholic fatty liver disease (NAFLD). From the pool of genes sensitive to nutrient content, we selected genes characterized by a strong response to the NIOR. The polymorphisms included Adrenergic receptor (*b3AR*), Tumor necrosis factor (*TNFα*), Apolipoprotein C (*Apo C III*). Uncoupling Protein type I (*UCP-1*), Peroxisome proliferator activated receptor γ2 (*PPAR-2*) and Apolipoprotein E (*APOEs*). We performed three dietary interventions: a diet consistent with the results of genotyping (NIOR (+)); typical dietary recommendations for NAFLD (Cust (+)), and a diet opposite to the genotyping results (NIOR (−) and Cust (−)). We administered the diet for six months. The most beneficial changes were observed among fat-sensitive patients who were treated with the NIOR (+) diet. These changes included improvements in body mass and insulin sensitivity and normalization of blood lipids. In people sensitive to fat, the NIOR seems to be a useful tool for determining specific strategies for the treatment of NAFLD.

## 1. Introduction

Non-alcoholic fatty liver disease (NAFLD) is one of the most frequently diagnosed liver diseases in the industrialized world—approximately 20%–30% of nations’ populations are affected by it [1,2]. With the increase in obesity, NAFLD has become a major risk factor for cirrhosis (and other diseases, e.g., cardiovascular diseases) [3]. Multiple trials have demonstrated that weight loss reduces histological steatosis (intrahepatic fat content) and the amount of serum enzymes [4].

One of the key causes of NAFLD is an improper diet based on caloric oversupply, the excessive intake of fats, and, at the same time, the low intake of grains, fruits, vegetables, proteins and ω-3 fatty acids [2]. This pattern of nutrition leads to the development of hyperinsulinemia, insulin resistance and obesity [2,5,6,7]. Therefore, on the one hand, nutrition is a major cause of NAFLD, but on the other, it presents an effective form of treatment [5,8,9].

In NAFLD, nutrition can be characterized by an appropriate choice of active nutrients that can play a regulatory role in metabolism. Nutrients regulate the activity of enzymes involved in metabolic processes, acting at the level of the proteome and metabolome and functioning as sensors that influence metabolic pathways [10,11,12]. Importantly, the same nutrient may have different influences on given people due to genetic polymorphisms found in the population [12]. The interactions between nutrients, genetic factors (polymorphism/mutations) and health are the subject matter of nutrigenomics [12]. This field of science aims to establish personalized nutrition strategies for the prevention and treatment of lifestyle diseases [12,13]. It can be assumed that if the action of nutrients is affected by polymorphisms, it is advisable to search for methods of individualizing a patient’s nutrition. Therefore, in this study, we focused on testing a tool that could be used for the individualization of nutrition in patients with NAFLD. The specific tool used in this study was the nutrient-induced insulin output ratio (NIOR), which was selected to determine the genotype-phenotype interaction [14]. The NIOR has already been used to identify a correlation between the variants of genes (associated with the metabolism of carbohydrates and fat) and the output of insulin and the development of diet-induced insulin resistance. Using the NIOR, we identified the carriers of the alleles of gene variants characterized by a reduced tolerance to fat or carbohydrates in the diet. The pool of genes associated with NIOR includes glucose-sensitive genes, such as genes for Adrenergic receptors (*b3AR*), Tumor necrosis factor (*TNF-α*) and Apolipoprotein C (*APOC*3) [14]. The variants of these genes are described in the literature as being responsible for an increased risk of developing insulin resistance (gene *b3AR*, rs 4994) [15], the induction and development of insulin resistance and metabolic syndrome (gene *TNF-α*, rs 1800629) [16] and severe forms of hyperlipidemia (gene *APOC3*, rs 5128) [17].

Fat-sensitive genes associated with NIOR include the genes of Uncoupling Protein type I (*UCP-1*, rs 1800592), Peroxisome proliferator-activated receptor γ 2 (*PPAR-γ2*, rs 18012820) and Apolipoprotein E (*ApoE*). Selected variants of these genes are responsible for the regulation of body weight and the concentration of plasma high density lipoprotein (Type 1 uncoupling protein (*UCP1*)) [18], an increased risk of metabolic syndrome by the regulation of energy homeostasis and glucose (Peroxisome proliferator activated receptor γ2 *PPAR-γ2* gene) [19], the furthering of insulin-resistance, the development of hyperlipidemia and hypertriglyceridemia and the progression of coronary heart disease (*APOE* rs 405509, rs 7412 rs 429358) [17].

The aim of this study was to determine whether the NIOR can be useful in planning the individualized nutrition of patients with NAFLD and whether its use contributes to a more effective inhibition of NAFLD progression, defined as a reduced degree of hepatic steatosis and improved biochemical and anthropometric parameters.

## 2. Results

### 2.1. The Analysis of the Data Using Model 1

#### 2.1.1. Changes in Anthropometric Parameters after Six Months Depending on the Type of Diet

The most beneficial changes in body composition were observed among patients treated with the NIOR (+) diet (Table 1). The body mass reduction, the reduction in waist circumference, and the reduction in fat mass were significant.

Weight reductions were also recorded in the Cust (+) group, but in comparison to NIOR (+), the reduction in fat content was less significant (−3.40 ± 6.27, *p* < 0.002 vs. −0.66 ± 3.67, *p* < 0.02) (Table 1). In Cust (+) patients, negative changes associated with the loss of lean body mass and arm circumference were also recorded (Table 1).

Slight changes in body mass, waist circumference, and hip circumference were observed in the group contrary to NIOR (−) and Cust (−) (called CONTRA in Table 1).

The analysis of changes between these groups provided interesting results. The most significant changes were observed when NIOR (+) and NIOR (−) and Cust (−) were compared (CONTRA NIOR (−) and Cust (−)). Between these groups, there were significant differences in the reduction of body mass (−6.79 ± 4.79 kg, NIOR (+) vs. −2.56 ± 2.88 kg NIOR (−), *p* < 0.026), BMI (−2.41 ± 1.73 kg/m^2^ NIOR (+) vs. −0.83 ± 1.04 kg/m^2^ NIOR (−), *p* < 0.015), fat mass (−5.39 ± 6.19, *p* < 0.006 NIOR (+) vs. −0.136 ± 2.97 NIOR (−), *p* < 0.007) and fat content (−2.45 ± 7.01 NIOR (+) vs. −0.88 ± 3.00 NIOR (−), *p* < 0.005) (Table 1).

Between the groups Cust (+) and NIOR (+), we observed a significant difference in the reduction of fat mass (−3.40 ± 6.27 kg Cust (+) vs. −5.39 ± 6.19 kg NIOR (+), *p* < 0.04) (Table 1).

Between the groups Cust (+) and Cust (−), we found a difference in arm circumference change (−1.45 ± 1.60 cm Cust (+) vs. 1.05 ± 3.01 cm Cust (−), *p* < 0.04) (Table 1).

#### 2.1.2. Changes in Biochemical Parameters after Six Months in Model 1

One of the most important objectives to achieve during nutritional therapy in patients with NAFLD is a reduction in insulin resistance [20]. This effect was measured by determining. The homeostatc model assessment HOMA IR and HOMA B (used to estimate the improved β-cell “function”) [21]. HOMA IR under normal physiological conditions is 1.0; higher values indicate peripheral insulin resistance or resistance of hepatic origin [22,23]. Patients in all groups were characterized by insulin resistance at the beginning (Table 1). The highest average HOMA IR value was observed for the NIOR (−) and Cust (−) groups. The reduction in HOMA IR in both of these groups reached −2.64 ± 4.57, *p* < 0.05. The initial HOMA IR in NIOR (+) patients was 3.76 ± 1.94. The recorded reduction in HOMA IR after six months was −1.34 ± 1.86, *p* < 0.05 (Table 1).

Additionally, the normalization of blood lipids (total cholesterol, triglyceride (TG), low density lipoprotein (LDL), high density lipoprotein (HDL) is an important element of nutritional therapy. Positive trends toward blood lipid normalization were observed in all types of diets (Table 1).

#### 2.1.3. A Significant Reduction in the Degree of Fatty Liver Disease Was Observed in Patients with a Diet Selected According to NIOR

In the NIOR (+) group, the average reduction in the degree of fatty liver disease was −1.31 ± 1.01, *p* < 0.002. The difference in the reduction of fatty liver disease was significant between the NIOR (+) and NIOR (−) groups, *p* < 0.04—Mann-Whitney *U* test (Table 1).

### 2.2. The Data Analysis in Model 2

Individuals from Different Groups Who Had a Similar Range of Reduction in Body Weight Obtained Different Reductions in Hepatic Steatosis and Other Parameters

**Figure 1:**
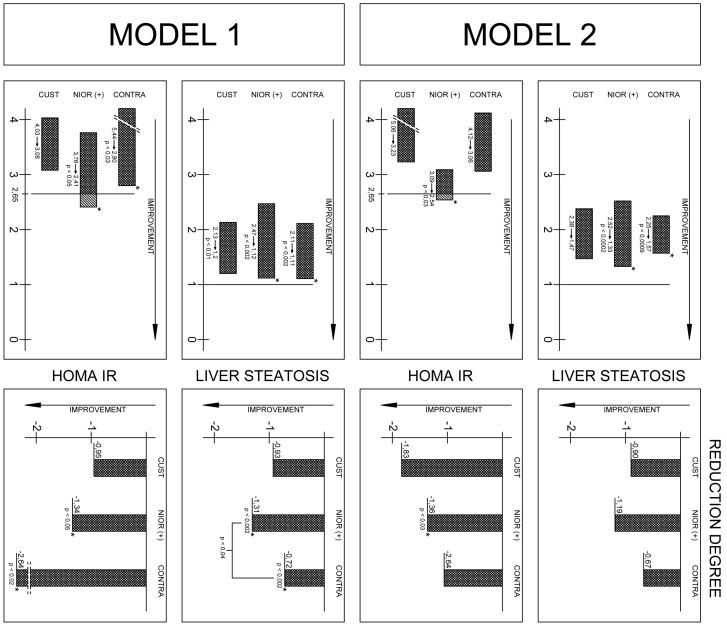
Changes in biochemical blood parameters in Model 1 and 2Note: All data represent the mean (standard deviation).

Only individuals in the NIOR (+) group showed improvement in the degree of hepatic steatosis (Figure 1, Table S1).

The analysis of the differences between the groups showed that the reduction in hepatic steatosis in the NIOR (+) group significantly differed from that observed in the NIOR (−) group (Mann-Whitney *U* test, *p* < 0.04). A similar significant difference between groups was observed for hyaluronic acid, with levels differing significantly between the Cust (+) and NIOR (+) groups (−26.45 ± 17.72 NIOR (+) vs. −1.94 ± 5.4 Cust (−) and NIOR (−), Mann-Whitney *U* test, *p* < 0.005).

## 3. Discussion

Obesity and insulin resistance present a considerable challenge in the nutrition plans of patients with NAFLD [24,25]. Current dietary guidelines are based on epidemiological data showing a link between diets enriched in saturated fatty acids and in fructose and the development of insulin resistance [26]. However, the response to diet differs depending on individual variations in genetic and metabolic phenotypes. Therefore, it is important to personalize patients’ diets, taking into account their genetic predispositions [13,14].

One potentially interesting tool is the nutrient-induced insulin output ratio (NIOR). The NIOR makes it possible to categorize patients (gene variant carriers) into two groups: phenotypically sensitive to glucose or fat in the diet. The polymorphisms of genes associated with the NIOR have previously been associated with the severity of metabolic syndrome and susceptibility to the effects of nutrients [14,15,16,17,18,19]. In our work, we examined polymorphisms (linked with NIOR) according to their impact on the output of insulin after a meal [14]. The usefulness of NIOR as a potential tool to individualize diets was examined through the introduction of quantitative changes in nutrients (fat or simple carbohydrates), consistent with the results of genetic tests. To exclude the impact of polymorphisms themselves, some people were randomly assigned to a group in which the key nutrient contents were chosen in quantities contrary to the indications of genetic research.

The second important objective of this study was to create a nutritional plan that would be accepted by the respondents for an extended period of time. We succeeded in obtaining the results of a half-year-long diet, resulting in an acceptable reduction in the content of the tested nutrients in the diet.

We showed that a selection of nutrients consistent with the indications of the NIOR contributed to an effective reduction in hepatic steatosis in both Model 1 and Model 2. This is a very important result, as fat droplets accumulating in hepatocytes are considered the main hepatotoxic factor, inducing hepatic steatosis and fibrosis [27,28,29]. The reduction of lipid content in the liver, therefore, means a reduction in the intensity of fibrosis [27], which is marked by hyaluronic acid content in the blood [28,29]. Such a reduction in hyaluronic acid was recorded in all groups, but the largest decline in hyaluronic acid content was found in the NIOR (+) group, regardless of the research model (Table 1 and Figure 1).

Additionally, individual selections of nutrients based on the NIOR were intended to contribute to the reduction of fat mass (Table 1). The results seem to confirm the usefulness of NIOR for the efficient reduction in body fat mass and fat content when comparing the NIOR (+) and NIOR (−) groups. It seems that the reduction of fat mass and fat tissue was most effective in the group in which the amount of dietary fat or dietary sugar was adjusted to gene polymorphisms. Of note is that there was no significant effect of NIOR on the reduction of insulin resistance between groups. The HOMA IR ratio was effectively reduced in all groups, regardless of the type of diet (Table 1). Fats are components that play a crucial role in the progression of NAFLD [27,30,31,32,33,34]. The positive changes in the liver were the result of a decrease in the fat content of the diet, especially among fat-sensitive polymorphism carriers (Table 1 and Figure 1). Our study confirms the results of other authors, e.g., Marina et al. [31], who found that fat (in different contents in the diet—20% vs. 55% the total daily energy expenditure (TDEE) caused minor effects in the content of intra-abdominal fat and intrahepatic lipids. In another study (a short-term intervention), a three-week isocaloric low-fat diet (20% TDEE ) decreased intrahepatic lipids by 13%, whereas a high fat diet (55% TDEE) increased the amount of lipids in the liver by up to 17% [30]. Unfortunately, both studies were limited to a short period of observation [30,31].

**Figure 2:**
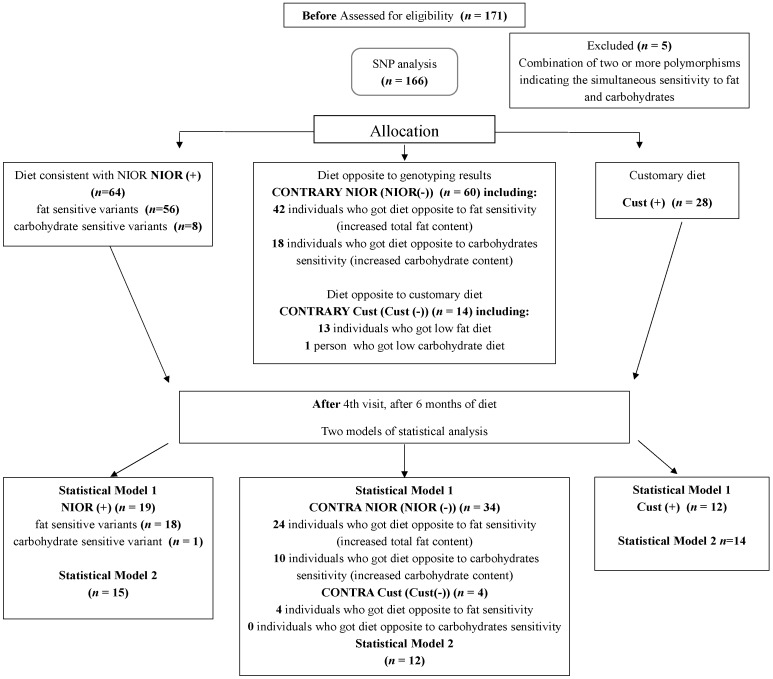
Flowchart for the selection of individuals from the nutrient-induced insulin output ratio (NIOR) cohort. Participants entering subsequent phases of the study as well as dropouts out are indicated in the total. NIOR (+) represents individuals consuming a diet consistent with the results of genotyping; Cust (+), individuals consuming a diet comprising the typical dietary recommendations for non-alcoholic fatty liver disease (NAFLD); NIOR (−) and Cust (−), individuals consuming a diet contrary to the genotyping results.

Though our study was longer, it suffered from a significant limitation, which was the exclusion of variants sensitive to simple sugars (after six months, only one person remained—Figure 2). This was a substantial loss because simple sugars, especially fructose (a common nutrient in western diets), is reported to be associated with an increased risk of NAFLD [35,36,37]. Although the consumption of fructose is high and continues to be on the rise [38], there are still no conclusive results that indicate a connection between the high intake of fructose and NAFLD [35,37]. The available evidence is not sufficiently robust to draw conclusions regarding the effects of fructose, high fructose corn syrup (HFCS) or sucrose consumption on NAFLD [37].

It seems that the lack of clear associations between the consumption of simple sugars and hepatic steatosis can result from yet another important variable, i.e., gender. Research from 2014 shows that the severity of hepatic steatosis may be significantly influenced by feeding patterns associated with gender [27]. Unfortunately, our study cannot be included in the discussion in this area. Slightly more severe hepatic steatosis was shown in our analysis of diets before the initiation of the prescribed diet. The analysis of the FFQ results indicates a lack of a relationship between the consumption of products containing large amounts of sugars and the degree of hepatic steatosis among our respondents, regardless of gender (unpublished results). Understanding the specific interaction between nutrients and dietary needs and maintaining this balance is extremely important in providing treatment for NAFLD [39,40].

## 4. Materials and Methods

### 4.1. Patients

A group of 171 eligible participants, Caucasian men (*n* = 104) and women (*n* = 67) diagnosed with NAFLD, were prospectively enrolled in the study (Figure 2). Of the 171 total recruited patients, only 166 confirmed patients with NAFLD met the inclusion criteria. We conducted the measurements at the beginning of the study and at check points conducted at the first visit, after the first month, the second month and after six months—the final check point (Figure 2).

The exclusion criteria included the following: diabetes mellitus (DMII); infection with either HBV (Hepatitis B Virus) or HCV (Hepatitis C Virus); obesity (body mass index (BMI) >30 kg/m^2^); high levels of physical activity (>3000 kcal/week in leisure-time physical activity); changes in physical activity during the dietary intervention; use of statins; any condition that could limit the mobility of the participant; not being able to attend control visits; vegetarianism or a need for other special diets; the excessive consumption of alcohol (≥20 g in women and ≥30 g in men, per day); and other drug addiction.

Physical activity was assessed during the first visit and in subsequent appointments using the International Physical Activity Questionnaire (IPAQ) [41]. In this study we recommended moderate activity and we advised our patients not to change physical activity during the time of intervention. The degree of fatty liver disease was assessed by a trained physician according to the Hamaguchi score [42], using a high-resolution B-mode abdominal ultrasound scanner (Acuson X300, Simens, San Jose, CA, USA).

The study protocol was approved by the ethics committee of the Pomeranian Medical University (Szczecin, Poland, 25 01 2010 KB-0012/09/10) and conformed to the ethical guidelines of the 1975 Declaration of Helsinki. The volunteers provided written informed consent before the study.

### 4.2. The Anthropometric Data

Anthropometric assessments were performed routinely during each of the four visits. The study included measurements of height (m), body weight (kg), skinfold thickness (mm), arm circumference (cm), waist circumference (cm) and hip circumference (cm). The measurements of body weight and height were obtained by means of medical scales with a stadiometer. Body mass index was calculated according to these measurements (BMI = body weight (kg)/square of height (m)) [24,43]. Using a medical tape measure, waist circumference was measured (midway between the bottom edge of the ribs and the iliac crest) as was hip circumference. Based on these measurements, WHR was calculated (WHR = waist circumference (cm)/hip circumference (cm)) [24]. A caliper was used to measure skinfold thicknesses: biceps, triceps, subscapular and abdominal skinfolds. In addition, in each subject, body composition was measured with a multifrequency bioimpedance meter, BIA-101 (Akern, Bioresearch SRL, PONASSIEVE, Florence, Italy).

### 4.3. Methods and Experimental Design

**Figure 3:**
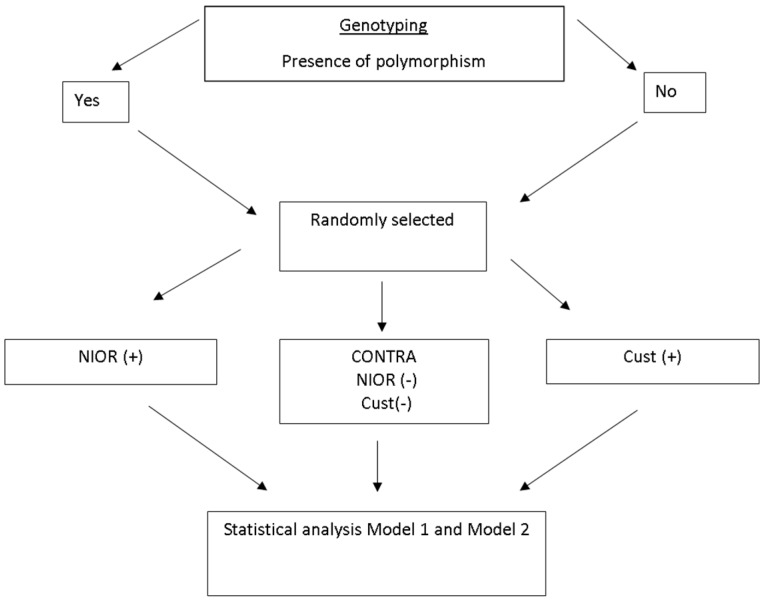
Baseline treatment characteristics. NIOR (+) represents individuals consuming a diet consistent with the results of genotyping; Cust (+), individuals consuming a diet comprising the typical dietary recommendations for NAFLD; NIOR (−) and Cust (−), individuals consuming a diet contrary to the genotyping results.

A randomized parallel controlled clinical trial with three dietary interventions was performed:

1. A diet consistent with the results of genotyping, called NIOR (+);
2. A diet with typical dietary recommendations for NAFLD, called Cust (+) [8];
3. A diet opposite to genotyping results, called (NIOR (−) and Cust (−) (CONTRA NIOR and Cust) (Figure 3).

### 4.4. Allocation to Groups

The patients were randomly assigned to the NIOR (+) group. They represented:

(a) a single polymorphism indicative of sensitivity to carbohydrates or fats
(b) more than one polymorphism indicative of sensitivity to carbohydrates or to fats (e.g., two polymorphisms indicative of sensitivity to fat)

Only eight patients from the NIOR (+) group had polymorphisms indicative of sensitivity to carbohydrates. Unfortunately, these people dropped out of the study at various stages of the study. Only one carrier of sensitivity to carbohydrates completed the study (19 patients remained in the group).

Persons with a combination of two or more polymorphisms indicating simultaneous sensitivity to fat and carbohydrates were excluded from the study.

### 4.5. Dietary Intervention

#### 4.5.1. General Recommendation

The diet was calculated individually according to the patient’s caloric needs. Individuals with a BMI indicating that they were overweight or obese received a reduced caloric diet of 500 kcal/day. People with a BMI within the normal range were given a normocaloric diet that allowed them to maintain their current body weight.

The total daily energy expenditure (TDEE) was calculated using the direct measurement of resting metabolic rate (RMR). RMR was measured during the first visit and in subsequent follow-up visits with a Fitmate apparatus (Pro, COSMED). The activity factor (AF) was determined in accordance with the generally accepted norm (TDEE = AF × RMR). The caloric content of the diet was adjusted during visits to the changing values of the patient’s TDEE.

All patients received weekly menus and guidelines on the timing of meals throughout the day, their composition and the size of the portions. Menus were prepared in the form of a daily plan for the seven days of the week and included guidance on the timing during the day of the five meal times.

The recommended sources of fat included vegetable fats, with a predominance of rapeseed oil and olive oil. It was permissible to use butter and margarine. Animal fats such as lard were excluded.

The recommended sources of carbohydrates included products with a low and medium glycemic index (GI). These included whole wheat bread, whole wheat pasta, cereal and brown rice. Sweets were excluded from the diet.

The recommended protein sources comprised poultry, fish (oily fish three times a week), fermented dairy products (two times a day), eggs (four to five times a week), lean cottage cheese, and cheese with a reduced fat content. Pork fat and offal products were excluded from the diet. The amount of fruit and vegetables recommended in the diets included three portions of vegetables and two portions of fruit. The amount of fluid intake was calculated to be 35 mL/kg of actual body weight.

#### 4.5.2. Recommendations Based on the Nutrient-Induced Insulin Output Ratio (NIOR)

(a) NIOR (+) patients received dietary recommendations with a reduced fat content (20% TDEE when NIOR polymorphisms showed sensitivity to fat) or reduced carbohydrate content (55% of TDEE, including <5% of sugars, when the polymorphisms showed sensitivity to simple carbohydrates).
(b) Cust (+) patients received dietary advice with the following nutrient content: fat content at 30% of TDEE and carbohydrates at 55% of TDEE (including 10% of simple carbohydrates).
(c) NIOR (−) patients, when they had “fat-sensitive” gene variants, received dietary recommendations that increased total fat content up to 30% of TDEE.

When participants had sugar-sensitive variants of genes, they received an increased amount of carbohydrates (10% simple carbohydrates).

Cust (−) patients were randomly assigned to groups with a reduced fat content or lower carbohydrate content.

#### 4.5.3. Dietary Control

Nutrition patterns were analyzed with a Food Frequency Questionnaire (FFQ) and a 72 h food diary (including two working days and one day free of work) during the first visit. At all check points, the patients brought their completed 72 h food diary. The amounts consumed were recorded in household units, by volume or by measuring with a ruler. The dietary records were validated by a nutritionist according to a corresponding food table and nutrient database (Table 2).

### 4.6. Laboratory Analyses

After overnight fasting, venous blood was collected into tubes containing anticoagulant Ethylenediaminetetraacetic acid (EDTA).

Blood samples were centrifuged at 3500 rpm for 10 min at 4 °C within 2 h of collection. Standard blood biochemical analyses were carried out at the University Hospital Laboratory (Szczecin, Poland). Hyaluronic acid was determined with an ELISA kit (Wuhan EIAab Science, A1710 Guangguguoji, Wuhan, China).

### 4.7. Genotyping

From the pool of genes sensitive to nutrient content, we selected genes that were characterized by a strong response to the oral glucose tolerance test after 75 g of glucose or after a high-fat meal. These included the b3-adrenergic receptor (b3AR), tumor necrosis factor (TNF-α) and apolipoprotein C III (apo CIII) [14].

From the pool of carbohydrate-sensitive genes, we selected Type 1 uncoupling protein (UCP-1), peroxisome proliferator-activated receptor γ 2 (PPAR-Y2) and apolipoprotein E (ApoE).

DNA from mononuclear peripheral blood was isolated using a DNeasy Blood and Tissue kit (Qiagen, Valencia, CA, USA). Genotypes were determined by the real-time polymerase chain reaction using TaqMan^®^ Genotyping 36 g Assays for polymorphisms, including b3AR rs4994 (Applied Biosystems Assay ID C___2215549_20); TNF-rs1800629 (C___7514879_10); Apo C III-rs5128 (C___8907537_1); Ucp-1-rs1800592 (C___8866368_20); PPAR-2-rs 1801282 (C___1129864_10); APOE-rs 405509 (C____905013_10); APOE-rs7412 (C_904973_10); and APOE-rs429358 (C___3084793_20). Fluorescence data were analyzed with allelic discrimination—7500 Software v 2.0.2 (Foster City, CA, USA).

### 4.8. Statistical Analysis

Statistica 7.1 software (Statsoft, Poznań, Poland) was used for the statistical analysis, and all results are expressed as the mean ± standard deviation. As the distribution, in most cases, deviated from normal (Shapiro-Wilk’s test), non-parametric tests were used: Wilcoxon tests were used for comparisons among groups and Mann-Whitney *U* tests were used for comparisons between groups. A *p* < 0.05 was considered significant.

#### Two Models of Statistical Analysis

Model 1 included the analysis of the results of the anthropometric and biochemical measurements with the criterion of the dietary recommendations that were adopted by the patients throughout the study (six months). The caloric value of the patients’ menus was estimated during checkups, which took place after one, two and six months, based on their 72 h diaries. The patients who were included in the statistical analysis followed the diet carefully (which was estimated based on menus in relation to the recommended caloric content ±200 kcal/day). Patients who exceeded that value at any stage of the study were excluded from the statistical analysis in Model 1.

Model 2 included the analysis of the anthropometric and biochemical results with the criterion of weight loss in the range of 8–10 kg over six months. We excluded patients who were characterized by normal weight at the beginning of the experiment from this analysis.

## 5. Conclusions

It seems that by introducing an individual nutrition and genotyping plan that takes into account the normal supply of calories, nutrients, proteins, and micro- and macronutrients, we are able to prevent problems that result from the progression of disease. Therefore, individualization, understood as the work of a dietitian with the patient, seems to be a therapeutic necessity, and the nutrient-induced insulin output ratio in people sensitive to fat seems to be n useful tool for determining specific strategies for patients with NAFLD.

## Figures and Tables

**Table 1:** Anthropological and biochemical characteristics of the study participants’ blood parameters at baseline and after six months of the diet in Model 1, with *p*-values of the comparison between subjects within this same intervention before and after six months. ^a^ *p* < 0.0005 Wilcoxon test, comparison between baseline and the fourth visit in this same group; ^b^ *p* < 0.005 Wilcoxon test, comparison between baseline and the fourth visit in this same group; * Mann-Whitney *U* test, comparison between NIOR (+) and Cust (+); ^#^ Mann-Whitney *U* test, comparison between NIOR (+) and contrary diets NIOR (−)/Cust (−); ^&^ Mann-Whitney *U* test, comparison between Cust (+) and contrary diets Cust (−) and NIOR (−). BMI: Body mass index; MUFA: monounsaturated fatty acids; PUFA: polyunsaturated fatty acids, HA: hyaluronic acid.

Parameters	Baseline			24W			*p* Value
	CUST (+)	NOR (+)	CONTRA CUST (−) and NOR (−)	CUST (+)	NOR (+)	CONTRA CUST (−) and NOR (−)	
Age	52.12 ± 14.74	52.80 ± 12.37	51.87 ± 12.11	52.12 ± 14.74	52.80 ± 12.37	51.87 ± 12.11	
Body mass (kg)	94.70 ± 22.55 ^a^	89.01 ± 15.26 ^a^	92.20 ± 19.34 ^b^	87.59 ± 17.96 ^a^	82.21 ± 15.35 ^a,#^	89.63 ± 20.79 ^b,#^	^a^ *p* < 0.0005
							^b^ *p* < 0.005
BMI (kg/m^2^)	32.10 ± 4.13	30.70 ± 3.64	32.27 ± 6.59	30.01 ± 2.84	28.29 ± 15.35 ^#^	28.29 ± 15.35 ^#^	^#^ *p* < 0.015
Arm circumference (cm)	33.30 ± 3.64 ^a^	31.66 ± 2.87	32.33 ± 3.86	31.85 ± 2.56 ^a,&^	30.75 ± 3.46	33.38 ± 4.53 ^&^	^a^ *p* < 0.0005
							^&^ *p* < 0.04
Waist circumference (cm)	105.14 ± 14.69 ^a^	100.12 ± 11.75 ^b^	106.25 ± 14.45 ^b^	97.02 ± 10.63 ^a^	94.00 ± 11.97 ^b^	102.60 ± 16.02 ^b^	^a^ *p* < 0.0009
							^b^ *p* < 0.005
Hip circumference (cm)	105.14 ± 14.69 ^a^	100.12 ± 11.75 ^b^	106.25 ± 15.73 ^b^	104.20 ± 14.89 ^a^	94.00 ± 13.33 ^b^	102.60 ± 17.04 ^b^	^a^ *p* < 0.0009
							^b^ *p* < 0.005
Fat mass (%)	34.71 ± 5.78 ^b^	35.41 ± 5.67 ^a^	36.02 ± 14.09	31.31 ± 5.78 ^b,^*	29.74 ± 8.21 ^a,^*^,#^	35.09 ± 14.94 ^#^	^a^ *p* < 0.0006
							^b^ *p* < 0.002
							^#^ *p* < 0.007
							* *p* < 0.04
Fat content (%)	37.00 ± 6.63 ^b^	35.61 ± 10.96 ^b^	38.07 ± 7.79	36.34 ± 5.75 ^b,^*	31.03 ± 13.46 ^b,^*^,#^	38.95 ± 7.90 ^#^	^b^ *p* < 0.005
							^#^ *p* < 0.005
							* *p* < 0.04
Lean mass (%)	60.02 ± 16.47 ^b^	53.48 ± 10.96	55.94 ± 7.80 ^b^	55.92 ± 14.17 ^b^	51.03 ± 13.46	53.66 ± 9.28 ^b^	^b^ *p* < 0.002
AST (U/L)	36.10 ± 25.83 ^b^	30.70 ± 13.99 ^b^	32.71 ± 10.46 ^b^	35.10 ± 29.60 ^b^	34.20 ± 26.31 ^b,#^	23.85 ± 5.21 ^b,#^	^b^ *p* < 0.035
							^#^ *p* < 0.041
ALT (U/L)	54.00 ± 36.86 ^b^	46.70 ± 26.56 ^a^	47.92 ± 15.85 ^a^	44.40 ± 27.86 ^b^	39.40 ± 33.04 ^a^	32.57 ± 18.40 ^a^	^a^ *p* < 0.0006
							^a^ *p* < 0.038
Trigliceride (mg/dL)	129.30 ± 36.30 ^b^	123.80 ± 52.82	238.42 ± 482.09	106.40 ± 56.24 ^b^	121.80 ± 90.52	204.78 ± 319.17	^b^ *p* < 0.04
HDL (mg/dL)	51.00 ± 12.22 ^b^	54.40 ± 12.83	51.57 ± 15.73	54.00 ± 16.39 ^b^	54.30 ± 12.82	52.14 ± 14.31	^b^ *p* < 0.025
Insulin (mcU/L)	15.80 ± 9.05	14.59 ± 8.20 ^b^	18.54 ± 19.69 ^b^	12.07 ± 8.62	9.58 ± 6.81 ^b^	9.63 ± 9.46 ^b^	^b^ *p* < 0.05
HOMA–IR	4.03 ± 2.31	3.76 ± 1.94 ^b^	5.44 ± 6.65 ^b^	3.08 ± 2.08	2.41 ± 1.74 ^b^	2.80 ± 3.00 ^b^	^b^ *p* < 0.05
Hyaluronic acid (U/L)	54.56 ± 29.57	45.47 ± 25.82^b^	50.23 ± 31.76	45.93 ± 22.62	32.56 ± 16.28 ^b^	37.42 ± 22.21	^b^ *p* < 0.04
Fatty liver Hamaguchi score	2.13 ± 0.74 ^b^	2.47 ± 0.94 ^b^	2.11 ± 0.98 ^b^	1.2 ± 1.0 ^b^	1.12 ± 1.08 ^b,#^	1.11 ± 0.92 ^b,#^	^b^ *p* < 0.01
							^#^ *p* < 0.04

**Table 2:** Characteristics of dietary interventions. Nutrient-induced insulin output ratio (NIOR) (+) represents individuals consuming a diet consistent with the results of genotyping; Cust (+), individuals consuming a diet comprising the typical dietary recommendations for non-alcoholic fatty liver disease (NAFLD); NIOR (−) and Cust (−), individuals consuming a diet contrary to the genotyping results. * Group with a lower amount of carbohydrate (CHO) or fat.

Content of Diet	NIOR (+) Variant Sensitive for Fat	NIOR (+) Variant Sensitive for Carbohydrate	Cust (+)	CONTRA NIOR NIOR (−) If Variant Was Sensitive for Fat	CONTRA NIOR NIOR (−) If Variant Was Sensitive for Carbohydrate	CONTRA Cust Cust (−) Randomly Selected to Group with Lower Amount of Fat of CHO *
Energy	Calculated individually	Calculated individually	Calculated individually	Calculated individually	Calculated individually	Calculated individually
Fat percent of total caloric in %	20	30	30	30	20	20 or 30 *
Carbohydrates in %	65	55	55	55	65	65 or 55 *
Simple carbohydrate in %	≥10	<5	≥10	<5	≥10	≥10 or <5 *
Protein (%)	15	15	15	15	15	15
Fiber (g/day)	30–35	30–35	30–35	30–35	30–35	30–35
Fluid ( mL/kg)	35	35	35	35	35	35

## Supplementary Materials

Supplementary materials can be found at http://www.mdpi.com/1422-0067/17/7/1192/s1.
