# Supplementary material for: Nutritional Strategies for the Individualized Treatment of Non-Alcoholic Fatty Liver Disease (NAFLD) Based on the Nutrient-Induced Insulin Output Ratio (NIOR)
Source: Int J Mol Sci. 2016 Jul 22;17(7):1192. doi: 10.3390/ijms17071192 (PMC4964561; doi:10.3390/ijms17071192)
Supplement: Supplementary file 1 [file ijms-17-01192-s001.pdf]

Supplementary Materials: Nutritional Strategies for the Individualized Treatment of Non-Alcoholic Fatty Liver Disease (NAFLD) Based on the Nutrient-Induced Insulin Output Ratio (NIOR)

Ewa Stachowska, Karina Ryterska, Dominika Maciejewska, Marcin Banaszczak, Piotr Milkiewicz, Małgorzata Milkiewicz, Izabela Gutowska, Piotr Ossowski, Małgorzata Kaczorowska, Dominika Jamioł-Milc, Anna Sabinicz, Małgorzata Napierała, Lidia Wądołowska and Joanna Raszeja-Wyszomirska

Table S1. Changes in anthropological and biochemical blood parameters in Model 2. [a] $p < 0.0005$ Wilcoxon test; [b] $p < 0.005$ Wilcoxon test; [#] NIOR (+) consistent vs. contrary diets NIOR (−)/Cust (−). Mann-Whitney *U* test.

Parameters	Baseline			24W			*p* Value
	CUST (+)	NOR (+)	CONTRA CUST (−) and NOR (−)	CUST (+)	NOR (+)	CONTRA CUST (−) and NOR (−)	
Age	49.95 ± 12.32	47.90 ± 11.72	47.82 ± 13.08	49.95 ± 12.32	47.90 ± 11.72	47.82 ± 13.08	
Body mass (kg)	92.27 ± 23.44 [a]	86.99 ± 15.76 [a]	83.89 ± 16.13 [a]	80.36 ± 14.22 [a]	79.62 ± 13.12 [a]	77.99 ± 13.51 [a]	[a] $p < 0.0005$
BMI (kg/m^2)	30.51 ± 5.58 [a]	29.00 ± 3.94 [a]	27.54 ± 3.99 [a]	26.47 ± 3.15 [a]	26.28 ± 2.79 [a,#]	25.58 ± 2.74 [a,#]	[a] $p < 0.0005$<br>[#] $p < 0.04$
Arm circumference (cm)	32.16 ± 4.1	30.83 ± 3.24	30.19 ± 3.91	30.44 ± 2.91	29.49 ± 2.94	30.19 ± 4.41	
Waist circumference (cm)	101.01 ± 15.44	98.01 ± 10.96	97.13 ± 11.00	85.42 ± 26.73	89.87 ± 10.04	89.83 ± 9.01	
Hip circumference (cm)	103.16 ± 10.23	105.66 ± 6.87	101.02 ± 7.09	101.66 ± 8.02	101.26 ± 5.18	97.50 ± 5.90	
Fat mass (%)	32.66 ± 11.52	31.29 ± 10.02	26.46 ± 6.8	25.46 ± 7.37	25.7 ± 6.40	24.36 ± 4.14	
Fat content (%)	34.96 ± 5.96	36.1 ± 8.17	31.29 ± 2.93	31.58 ± 5.06	32.34 ± 5.21	31.63 ± 3.22	
Lean mass (%)	59.63 ± 13.64	55.22 ± 11.02	57.40 ± 9.98	54.89 ± 9.58	51.97 ± 11.90	53.21 ± 10.19	
AST (U/L)	27.73 ± 9.56	25.86 ± 8.74	44.33 ± 29.24	21.50 ± 4.94	20.20 ± 6.29	51.25 ± 94.91	
ALT (U/L)	44.71 ± 22.40 [b]	44.60 ± 24.95 [b]	64.33 ± 44.08 [b]	30.21 ± 14.20 [b]	26.93 ± 18.18 [b]	42.33 ± 39.94 [b]	[b] $p < 0.005$
Triglycerides (mg/dL)	123.50 ± 43.01	122.73 ± 56.95	143.00 ± 108.07	98.50 ± 49.14	134.06 ± 113.18	156.17 ± 99.73	
HDL (mg/dL)	47.57 ± 11.83	53.40 ± 13.34	53.33 ± 26.90	52.71 ± 15.26	52.00 ± 14.30	54.58 ± 20.09	
Insulin (mcU/L)	12.56 ± 14.99	11.62 ± 10.42	11.45 ± 6.75	7.96 ± 7.60	6.193 ± 6.162	6.19 ± 4.03	
HomaIR	3.57 ± 5.90	3.01 ± 2.87 [b]	2.84 ± 1.71	2.04 ± 2.02	1.55 ± 1.60 [b]	1.52 ± 1.03	[b] $p < 0.005$
Hylauronic Acid (U/L)	56.43 ± 22.40	51.28 ± 27.76 [b]	42.64 ± 19.98	38.91 ± 11.74	31.66 ± 16.44 [b]	36.69 ± 14.33	[b] $p < 0.005$
Fatty liver (Hamaguchi score)	2.38 ± 1.47	2.52 ± 0.87	2.25 ± 0.84	1.47 ± 0.92	1.33 ± 0.96 [a,#]	1.57 ± 1.03 [a,#]	[a] $p < 0.0005$<br>[#] $p < 0.04$
